# The non-specific lipid transfer protein McLTPII.9 of *Mentha canadensis* is involved in peltate glandular trichome density and volatile compound metabolism

**DOI:** 10.3389/fpls.2023.1188922

**Published:** 2023-05-31

**Authors:** Qiutong Chen, Li Li, Xiwu Qi, Hailing Fang, Xu Yu, Yang Bai, Zequn Chen, Qun Liu, Dongmei Liu, Chengyuan Liang

**Affiliations:** ^1^ Jiangsu Key Laboratory for the Research and Utilization of Plant Resources, Institute of Botany, Jiangsu Province and Chinese Academy of Sciences (Nanjing Botanical Garden Mem. Sun Yat-Sen), Nanjing, Jiangsu, China; ^2^ College of Forestry, Nanjing Forestry University, Nanjing, Jiangsu, China

**Keywords:** mint, nsLTPs, peltate glandular trichomes, violate oil, monoterpenes

## Abstract

*Mentha canadensis* L. is an important spice crop and medicinal herb with high economic value. The plant is covered with peltate glandular trichomes, which are responsible for the biosynthesis and secretion of volatile oils. Plant non-specific lipid transfer proteins (nsLTPs) belong to a complex multigenic family involved in various plant physiological processes. Here, we cloned and identified a non-specific lipid transfer protein gene (*McLTPII.9*) from *M. canadensis*, which may positively regulate peltate glandular trichome density and monoterpene metabolism. *McLTPII.9* was expressed in most *M. canadensis* tissues. The GUS signal driven by the *McLTPII.9* promoter in transgenic *Nicotiana tabacum* was observed in stems, leaves, and roots; it was also expressed in trichomes. *McLTPII.9* was associated with the plasma membrane. Overexpression of *McLTPII.9* in peppermint (*Mentha piperita.* L) significantly increased the peltate glandular trichome density and total volatile compound content compared with wild-type peppermint; it also altered the volatile oil composition. In *McLTPII.9*-overexpressing (OE) peppermint, the expression levels of several monoterpenoid synthase genes and glandular trichome development-related transcription factors—such as limonene synthase (*LS*), limonene-3-hydroxylase (*L3OH*), geranyl diphosphate synthase (*GPPS*), *HD-ZIP3*, and *MIXTA*—exhibited varying degrees of alteration. *McLTPII.9* overexpression resulted in both a change in expression of genes for terpenoid biosynthetic pathways which corresponded with an altered terpenoid profile in OE plants. In addition, peltate glandular trichome density was altered in the OE plants as well as the expression of genes for transcription factors that were shown to be involved in trichome development in plants.

## 1 Introduction

*Mentha canadensis* L., a member of the Lamiaceae family, is a widely cultivated spice crop and medicinal herb that is rich in volatile oil. The volatile oil in mint consists of monoterpenes (e.g., menthol, menthone, isomenthone, and pulegone etc.) and a small amount of sesquiterpenes that can be used as natural food additives, flavouring agents, and antioxidants (Wu et al., 2019; Do Nascimento et al., 2020; Karpiński, 2020; Mahendran and Rahman, 2020). Plant glandular trichomes are important places for the synthesis, secretion, and storage of specialised metabolites; they are regarded as biosynthesis factories (Huchelmann et al., 2017). Mint is densely covered with glandular trichomes, which comprise peltate and capitate trichomes. The volatile oil of mint was primarily produced and secreted by peltate glandular trichomes. The peltate glandular trichome consists of one basal cell, one stalk cell, and eight secretory cells (Turner et al., 2000). Mint volatile oil is produced by secretory cells and stored in peltate glandular trichomes within a subcuticular space that remains intact unless the leaf is damaged (Turner et al., 1999; Gershenzon et al., 2000; Tissier et al., 2017; Liu et al., 2023). The accumulation of volatile oil in peltate glandular trichomes is determined by secretory cell activity, volatile organic compound (VOC) transport, and storage cavity efficiency in terms of releasing VOCs into the atmosphere. However, the rate of VOC volatilisation is very low and does not significantly affect the accumulation of volatile oil in mint (Gershenzon et al., 2000; McConkey et al., 2000).

Transport of VOCs outside secretory cells requires plasma membrane-localised transporters, such as the adenosine triphosphate-binding cassette (ABC) (Adebesin et al., 2017), multidrug toxic compound extrusion (MATE), and non-specific lipid transfer proteins (nsLTPs) (Widhalm et al., 2015; Tissier et al., 2017). This selective transport of VOCs prevents cytotoxic effects related to excessive VOC accumulation (Eberl and Gershenzon, 2017; Gani et al., 2021).

nsLTPs are small basic proteins with hydrophobic cavities appropriate for binding and transporting lipids compounds, including fatty acids, acyl-coenzyme A, phospholipids, ceramide, and prostaglandin B2 (Edqvist et al., 2018; Salminen et al., 2018; Madni et al., 2020). The LTP family has numerous members. Boutrot et al. (2008) proposed a classification system based on a genome-wide analysis of rice, wheat, and *Arabidopsis thaliana*, which grouped nsLTPs into nine types (types I-IX) according to sequence similarities and Cys spacing. Liu et al. (2010) added type X. Edstam et al. (2011) studied the nsLTP subfamilies from early diverging land plants; they classified the nsLTPs into five major types (LTP1, LTP2, LTPc, LTPd, and LTPg) and five minor types (LTPe, LTPf, LTPh, LTPj, and LTPk). Analysis of the evolutionary relationships between nsLTPs in flowering and non-flowering plants revealed that nsLTPs are key proteins for plant survival and colonisation on land. During evolution, nsLTP family members were gradually selected and expanded; their biological functions became more complex (Edstam et al., 2011; Missaoui et al., 2022).

Plant nsLTPs contain an N-terminal secretory signal peptide that promotes proper subcellular localisation (Liu et al., 2015). nsLTPs are mainly present in the cell wall, plasma membrane, and intracellular components (Lee et al., 2009; Diz et al., 2011; Pagnussat et al., 2012; Deeken et al., 2016), presumably in relation to their functions. nsLTPs are implicated in the accumulation of cuticle wax, suberin, and sporopollenin; cell signalling; pollen and seed development; and cell expansion (Cameron et al., 2006; Kim et al., 2012; Champigny et al., 2013; Edstam et al., 2013; Tapia et al., 2013; Edstam et al., 2014; Liu et al., 2014). Additionally, some nsLTPs may be involved in metabolite synthesis and the secretion of glandular trichomes. For example, an NtLTP1-GFP fusion protein was abundant in lipids secreted from the long glandular trichomes of *NtLTP1-*overexpressing transgenic tobacco (Choi et al., 2012). Overexpression of *NtLTP1* in orange mint increased the diameter of peltate trichome heads and enhanced the emission of monoterpenes. Overexpression of *AaLTP3* and *AaLTP4* in transgenic *Artemisia annua* enhanced the production of sesquiterpene lactone (Adhikari et al., 2019). Overexpression of *BraLTP2* in *Brassica napus* led to increases in glandular trichome density and secondary metabolite content (Tian et al., 2018).

nsLTPs are highly expressed in mint glandular trichomes (Lange et al., 2000), and they may be involved in volatile oil transport and storage (Tissier et al., 2017). However, the biological functions of nsLTPs in mint are unclear. In this study, we characterised an nsLTP gene (*McLTPII.9*) from *M. canadensis*; we found that it was expressed in stems, leaves, and roots. McLTPII.9 was localised to the plasma membrane. Additionally, compared with wild-type (WT) peppermint, *McLTPII.9*-overexpressing peppermint (*Mentha piperita.* L) exhibit increased peltate glandular trichome density, along with alterations in volatile oil content and composition. Our results provide insight into the biological functions of nsLTPs in mint and will facilitate the breeding of new mint cultivars.

## 2 Materials and methods

### 2.1 Plant materials and growth conditions

The mint genotypes used in this study were *M. canadensis* and *M. piperita*, which are preserved in the Germplasm Resource Nursery of the Institute of Botany of Jiangsu Province and the Chinese Academy of Sciences. *M. canadensis* was used for gene (*McLTPII.9*) cloning, and *M. piperita* was used for mint genetic transformation. Shoot tips of mint plants were removed and transferred to one-quarter Hoagland’s culture medium until the roots grew out. Next, the mint plants were pot-cultured in nutrient-enriched soil under a 14-h light (26°C)/10-h dark (22°C) photoperiod until used.

The mint sterile explants were prepared as follows. Shoot tips of *M. piperita* surface sterilized for 10 min using 10% bleach and rinsed in 70% ethanol before being washed with sterile water three times. The explants were cultured in Murashige and Skoog (MS) medium with 2% sucrose (w/v) and 1% agar (w/v), and the pH was adjusted to 5.8. The sterile peppermint shoots were cultured at 26°C under a 14-h light/10-h dark photoperiod for further transformation experiments.

The tobacco genotypes used in this study were *Nicotiana tabacum* and *Nicotiana benthamiana*. *N. tabacum* was used for permanent genetic transformation, and *N. benthamiana* was used for transient transformation. The tobacco seedlings were grown in nutrient-enriched soil under a 16-h light (26°C)/8-h dark (24°C) photoperiod.

### 2.2 *In silico* characterisation of *McLTPII.9*

The coding sequence of *McLTPII.9* was polymerase chain reaction (PCR)-amplified from *M. canadensis* cDNA using the *F1* and *R1* primers (**Supplementary Table S1**), based on *M. canadensis* transcriptome data (Qi et al., 2018; Short Read Archive (SRA) Sequence Database accession number SRP132644). The National Centre for Biotechnology Information CD-Search tool (https://www.ncbi.nlm.nih.gov/Structure/cdd/wrpsb.cgi) was used to predict conserved domains in McLTPII.9. TMHMM 2.0 (https://services.healthtech.dtu.dk/service.php?TMHMM-2.0) was used to predict the transmembrane domain in McLTPII.9. The hydrophobicity of McLTPII.9 was analysed by ExPASy (https://www.expasy.org/). Signal peptide prediction in McLTPII.9 was performed using SignalP 6.0 (https://services.healthtech.dtu.dk/service.php?SignalP). For molecular modelling, a three-dimensional structural model of in McLTPII.9, along with four disulphide bridges in McLTPII.9, were predicted by Phyre2 (http://www.sbg.bio.ic.ac.uk/phyre2/html/page.cgi?id=index) and visualised in PyMOL software. Multiple sequence alignment and phylogenetic trees were constructed in MEGA v. 7.0 (maximum-likelihood, 1000 bootstraps, Poisson model). Gene coding and protein sequences are provided in Data sheet 1, and the sequences are available in the NCBI GenBank with accession number of OQ657221.

### 2.3 Vector construction and plant transformation

To generate the *McLTPII.9*-overexpression construct (p35S::McLTPII.9-GFP), the *McLTPII.9* coding sequence was PCR-amplified using primers *F2* and *R2*. The product was cloned into the modified pHellsgate8-GFP (p35S::GFP) vector using a recombinase (ClonExpress II One Step Cloning Kit, Vazyme Biotech, Nanjing, China). Mint plant transformation was performed by the *Agrobacterium tumefaciens*-mediated method (Niu et al., 1998; Qi et al., 2022; Yu et al., 2022). Briefly, the internode segments of sterile peppermints were used as explants and pre-cultured on pre-culture medium for 3 or 4 days. Then, the explants were immersed in the infection solution (*A. tumefaciens* containing recombinant plasmid resuspended in liquid MS medium) for 30 min. Remove the excess infection solution and dry the explants on sterile filter paper. The explants were cultured in co-cultivation medium for 4 days. Transfer the explants to the shoot induction medium with appropriate antibiotics for 4 weeks to generate resistant buds. Finally, resistant buds were transferred to a rooting medium for root generation.

The *McLTPII.9* upstream promoter sequence was amplified using the Genome Walking Kit (Takara, Dalian, China) with the *SP1*, *SP2*, and *SP3* primers. To generate the promoter_McLTPII.9_::GUS construct, a 1313-bp promoter sequence was amplified using the *Pro-McLTPII.9-F/Pro-McLTPII.9-R* primers, then cloned into the pMV2 plant binary vector (Hua et al., 2021). *N. tabacum* transformation was performed using the *A. tumefaciens*-mediated leaf disc method (Horsch et al., 1985). The primers are listed in **Supplementary Table S1**. The 1313-bp promoter sequence of McLTPII.9 is shown in Data sheet 1, and it is available in the NCBI GenBank with accession number of OQ658506.

### 2.4 Subcellular localisation of McLTPII.9

The *McLTPII.9*-overexpression plasmid (p35S::McLTPII.9-GFP) was used to assess subcellular localisation. The N-terminus of McLTPII.9 was fused to GFP under the control of the 35S promoter. A plasma membrane (PM) marker (AtCBL1n-mCherry) and a cell wall (CW) marker (AtLTPI.5-mCherry) were used as previously described (Deeken et al., 2016; Li et al., 2017). The recombinant vector and marker vectors were introduced into *A. tumefaciens* GV3101 for transient expression in tobacco (*N. benthamiana*); tobacco leaves were infiltrated as previously described (Li et al., 2017). Fluorescence was observed using an LSM 780 confocal microscope (Zeiss, Jena, Germany). The wavelength and emission bandwidth were for GFP as excitation 488 nm, emission 493–556 nm, and for mCherry as excitation 587, emission 570-690 nm. For plasmolysis, co-expression leaves were dipped in 0.8 M mannitol and observed after 15 min of incubation.

### 2.5 GUS staining

Histochemical staining of GUS expression in transgenic *N. tabacum* was performed in accordance with the method of Jefferson (1987). The roots, stems, and leaves were cut from the transgenic tobacco plants and were immersed in X-Gluc solution for incubating overnight at 37°C. After staining, the tissues were rinsed in 70% ethanol to remove chlorophyll.

### 2.6 Determination of peltate glandular trichome density

Peltate glandular trichomes on the leaf abaxial surface were observed using a stereo fluorescence microscope (Olympus, Tokyo, Japan) with a 1.6× objective at an excitation wavelength of 488 nm. The peltate glandular trichomes appear as fluorescent spots under stereo fluorescence microscope. The first, second, and third leaves of mint were used to count peltate glandular trichomes. The leaves of 3~5 plants with similar sizes were observed for each leaf position. Because the limited field of stereo fluorescence microscope, one snapshot does not capture an intact leaf. To get an image of the intact leaf, we captured multiple regions of a leaf, then spliced the images using Photoshop software. Finally, we count the number of peltate glandular trichomes and calculate density using these intact leaf images. The number of peltate glandular trichomes and leaf area were measured using ImageJ software.

### 2.7 Scanning electron microscopy

Mint samples were prepared as Qi et al. (2022) described. The second leaves of mint were cut into small pieces and fixed with 0.1 M phosphate-buffered 2.5% glutaraldehyde for 24 h at 4°C. Then the samples were washed with distilled water and dehydrated in an ethanol series. The samples were critical point dried and fixed on spherical metal stubs. The abaxial surface of leaves was coated with a thin layer of gold and observed using FEI Quanta 200 scanning electron microscope (FEI, Hillsboro, USA).

### 2.8 Analysis of volatile compounds

Leaves of transgenic and WT peppermint plants were carefully harvested and over-dried in the shade. For analysis of volatile compounds, 0.4 g of dry leaves were ground into powder and transferred to tightly closed 20-mL vials. Volatiles from these samples were extracted by headspace solid-phase microextraction, then measured by gas chromatography/mass spectrometry as described by Yu et al. (2021). The polydimethylsiloxane (PDMS) 65 µm fiber (65 µm Carboxen™-PDMS StableFlex, Supelco) was exposed to the headspace of the sample for 3 min at 40°C to adsorption. Then volatiles absorbed were desorbed in the injection port of GC at 250°C for 3 min.

### 2.9 RNA extraction and quantitative real-time PCR

Mint RNA extraction and RT-qPCR were performed as previously described (Li L, et al., 2020; Qi et al., 2022). The third and fourth leaves of mint plants growing for one month were used to isolate RNA. Leaves from the same position should preferably be in the same size. For qRT-PCR, 1μg of RNA was used for reverse transcription in a 20 μL reaction volume using the Prime Script™ RT Reagent kit with gDNA Eraser (TaKaRa, Dalian). The qRT-PCR amplification was performed using a qTOWER 2.2 Real-Time PCR system (Analytik Jena AG, Jena, Germany) with SYBR Universal qPCR Kit (Vazyme, Nanjing). The qRT-PCR conditions were as follows: 95 °C for 5 min followed by 40 cycles of 95°C for 15 s, 60°C for 15 s, and 72°C for 25 s. Relative expression levels were analysed by the 2^-ΔΔCT^ method. The primers for RT-qPCR are listed in **Supplementary Table S1**.

## 3 Results

### 3.1 Bioinformatic and phylogenetic analysis of the *McLTPII.9* gene and protein

**Figure 1:**
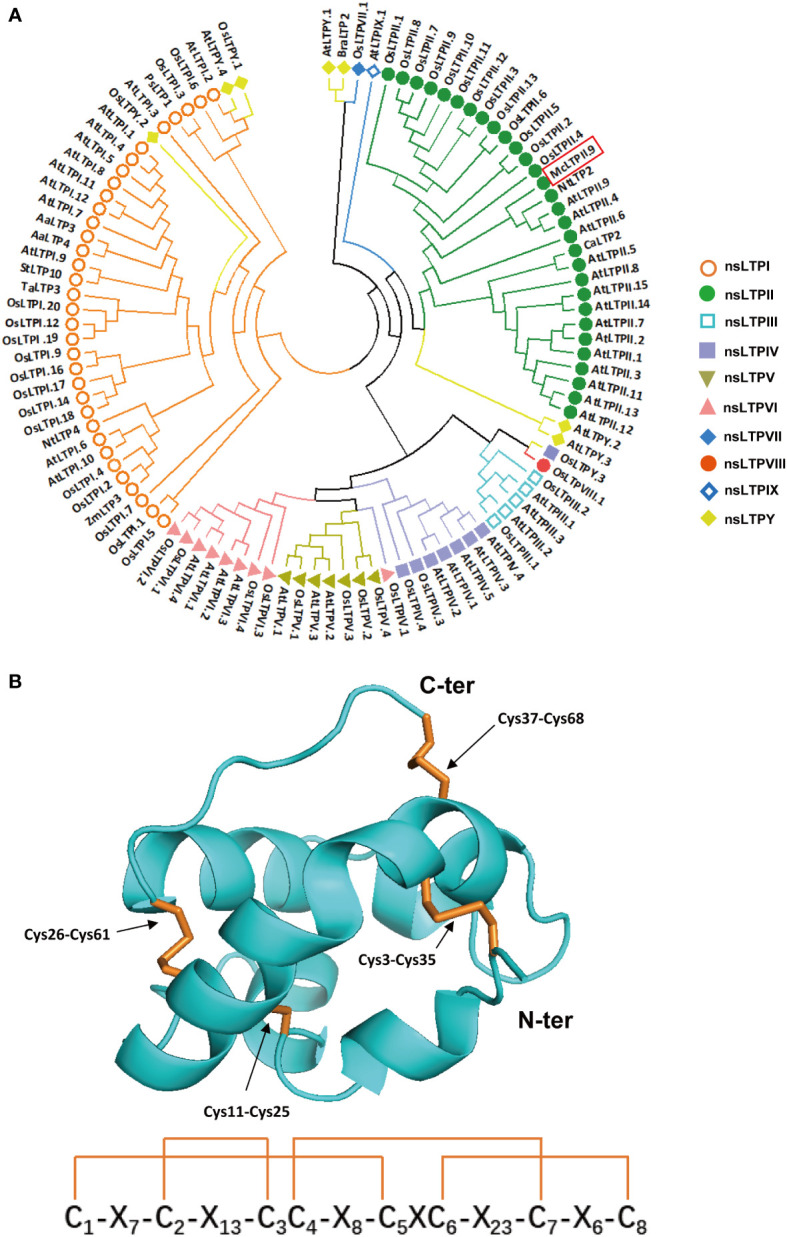
Phylogenetic analysis and three-dimensional modelling of McLTPII.9. **(A)** Phylogenetic analysis of McLTPII.9 and other nsLTPs from various plants. A maximum-likelihood tree was constructed using MEGA v. 7.0 (1000 bootstrap, Poisson model, partial deletion). nsLTPs were collected from *Arabidopsis thaliana, Oryza sativa, Nicotiana tabacum, Zea mays, Solanum tuberosum, Triticum aestivum, Artemisia annua, Coffea arabica, Pisum sativum*, and *Brassica rapa*, then classified into types I-Y (colours indicate types). Accession numbers are listed in **Table S2**. **(B)** Three-dimensional model of McLTPII.9. In the upper panel, the arrows point to the disulphide bridges of McLTPII.9. Cys, cysteine residue; C-ter, C-terminus; N-ter, N-terminus. The subscript represents amino acid site. The lower panel is a structure sketch of the four cysteine disulphide bonds referring to the upper panel. C1-C8 in the structure sketch correspond to Cys_3_, Cys_11_, Cys_25_, Cys_26_, Cys_35_, Cys_37_, Cys_61_, Cys_68_, respectively. X indicates other amino acid residues; the subscript represents the number of amino acids between adjacent cysteine residues. The orange linkages indicate disulfide bonds.

Previous studies suggested that nsLTPs were highly expressed in mint glandular trichomes, and they may be involved in volatile oil transport and storage (Lange et al., 2000; Tissier et al., 2017). So, in the beginning, we analysed the peltate glandular trichomes transcriptome data of *Mentha spicata* (Jin et al., 2014) for selecting nsLTPs with high expression levels in peltate glandular trichome, and performed local BLAST in *M. canadensis* transcriptome database (Qi et al., 2018; Yu et al., 2021). Finally, based on *M. canadensis* transcriptome data, we cloned an *McLTP*, which contained a 285-bp open reading frame encoding a protein of 94 amino acids. Amplification using the genomic DNA of *M. canadensis* as a template revealed that this gene had no introns. Conserved domains analysis of the amino acid sequence revealed that it was a type II nsLTP (**Supplementary Figure S1A**), which contains two adjacent hydrophobic cavities (Liu et al., 2015). Phylogenetic analysis of the McLTP revealed a close relationship to AtLTPII.9 and NtLTP2; therefore, we named the gene *McLTPII.9* (**Figure 1A**). Transmembrane domain and hydrophobicity analyses of McLTPII.9 showed that amino acids 5 to 29 were hydrophobic and constituted a transmembrane domain (**Supplementary Figures S1B, C**). McLTPII.9 was predicted to contain a signal peptide of 26 amino acid residues at the N-terminus. The predicted cleavage site was between amino acid residues 26 and 27 (**Supplementary Figure S1D**).

A molecular model of McLTPII.9 without a signal peptide was constructed using Phyre2 by homologous modeling method (**Figure 1B**) (Kelley et al., 2015). The most appropriate structural template for McLTPII.9 was LTP2G (PDB ID: tuka1). The proposed three-dimensional structure indicates that McLTPII.9 has typical LTPII features, including two adjacent hydrophobic cavities and four disulphide bonds formed by eight cysteine residues (Cys_3_-Cys_35_, Cys_11_-Cys_25_, Cys_26_-Cys_61_, and Cys_37_-Cys_68_) (**Figure 1B**, upper panel). The lower panel of **Figure 1B** is a structure sketch of the four cysteine disulphide bonds referring to the upper panel.

### 3.2 Expression and localisation of McLTPII.9

**Figure 2:**
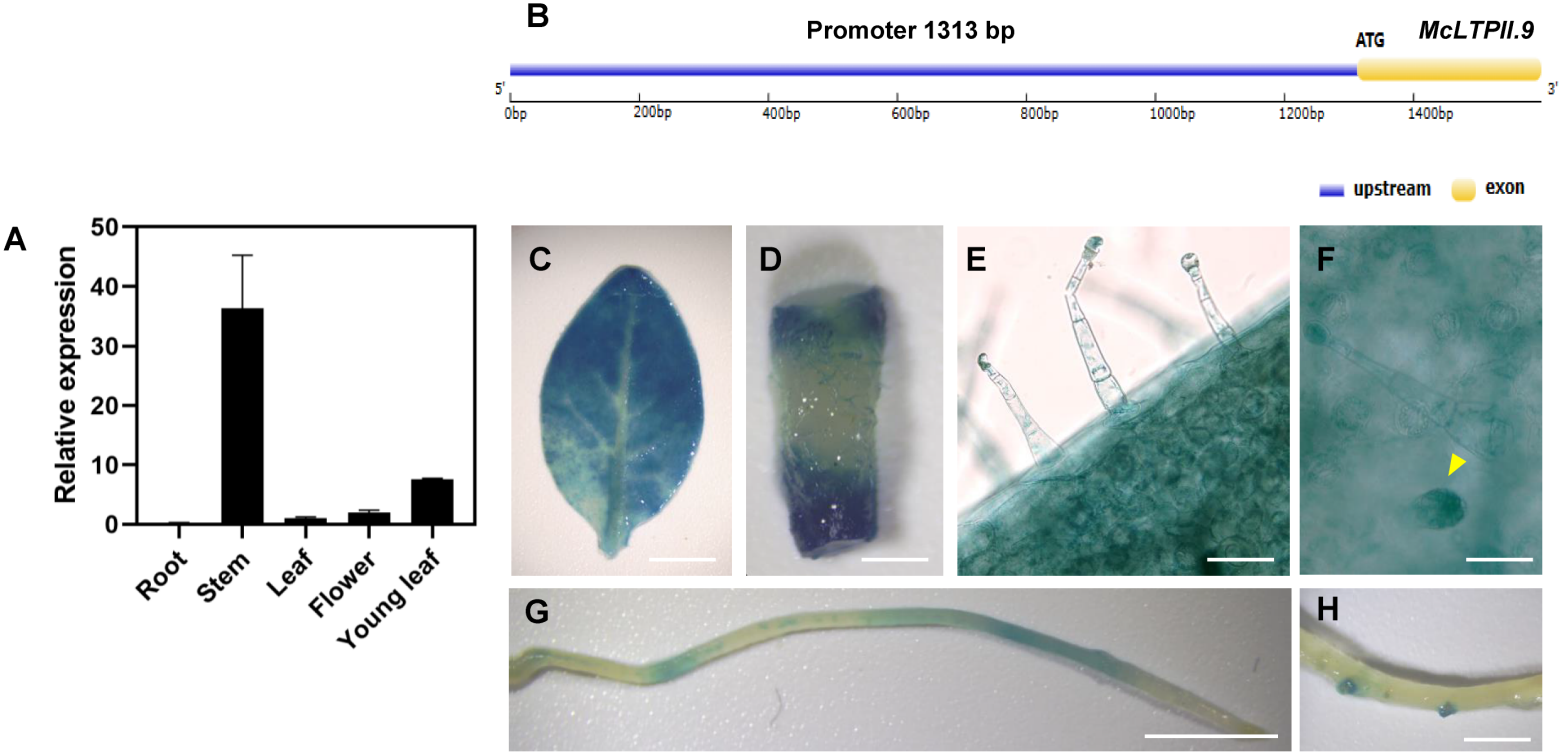
Expression pattern of *McLTPII.9*. **(A)** Tissue-specific expression analysis of *McLTPII.9* in *M. canadensis.* Data are means ± standard deviations of three biological replicates with three pooled tissues (plants) each. *MpActin1* was used as the internal control. **(B)** A gene model of *McLTPII.9* including the cloned promoter. **(C–F)** Histochemical GUS staining of transgenic *N. tabacum* (promoter_McLTPII.9_::GUS). Histochemical GUS-stained leaf **(C)**, stem **(D)**, long glandular trichomes **(E)**, short glandular trichomes (yellow arrow) **(F)**, and roots **(G, H)**. **(C, D, G, H)** Scale bars, 1 cm; **(E, F)** scale bars, 200 µm.

In *M. canadensis*, the expression of *McLTPII.9* was high in the stem and low in other part of the shoot and in roots (**Figure 2A**). In order to visualize *McLTPII.9* expression in plants, we transformed a GUS construct driven by the *McLTPII.9* native promoter (promoter_McLTPII.9_::GUS) into *N. tabacum*. The promoter of *McLTPII.9* was cloned from genomic DNA of *M. canadensis* by the genome-walking method. A gene model of *McLTPII.9* including the cloned promoter was shown in **Figure 2B**. In the transgenic *N. tabacum* plants, GUS-staining shows high staining intensity (presumably equaling expression) in leaves, medium in stems, and low in roots (**Figures 2C, D, G**). Specially, the staining was detected in lateral root primordia of transgenic *N. tabacum* (**Figure 2H**). In addition, the GUS signal was particularly in long and short glandular trichomes (**Figures 2E, F**); however, *McLTPII.9* expression in glandular trichomes were not tested independently in *M. canadensis*. In our results, two independent methods were used to test *McLTPII.9* expression in different plant species and that they lead to similar but not the same results.

**Figure 3:**
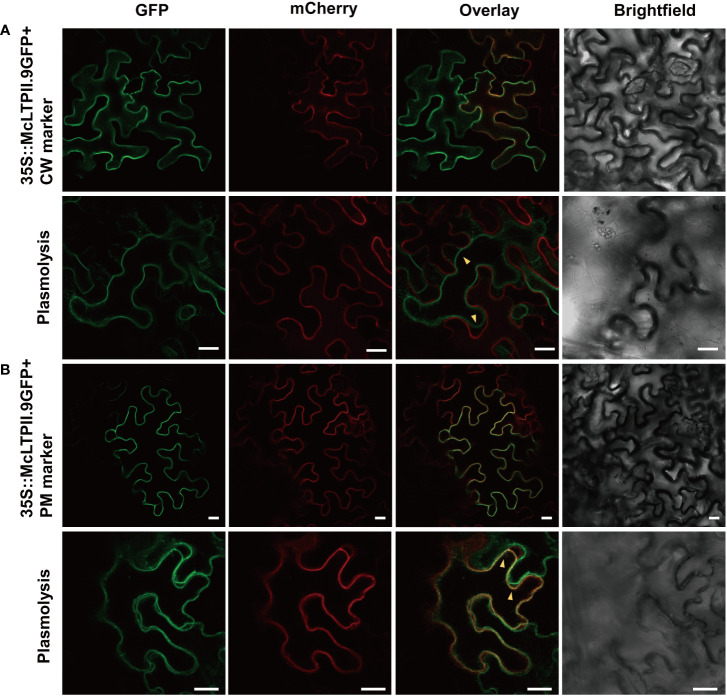
Subcellular localisation of McLTPII.9 in leaf epidermal cells of *N. benthamiana*. **(A)** Co-expression of the McLTPII.9-GFP fusion protein with a cell wall (CW; AtLTPI.5-mCherry) marker in tobacco epidermal cells. Lower row, plasmolysis assay with co-expressing tobacco leaves. Yellow arrows, separation between McLTPII.9-GFP and CW marker. Bars, 20 µm. **(B)** Co-expression of McLTPII.9-GFP fusion protein with a plasma membrane (PM; AtCBL1n-mCherry) marker in tobacco epidermal cells. Lower row, plasmolysis assay with co-expressing tobacco leaves. Yellow arrows, overlap between McLTPII.9 and PM marker. Bars, 20 µm.

To confirm the subcellular localisation of McLTPII.9, a McLTPII.9-GFP fusion protein was transiently co-expressed with a cell wall (CW) marker or a plasma membrane (PM) marker tagged with red fluorescent protein (mCherry) in *N. benthamiana* leaves. AtLTPI.5-mCherry (Deeken et al., 2016) served as the CW marker, and AtCBL1n-mCherry (Li et al., 2017) served as the PM marker. Confocal laser scanning microscopy showed similar superposition of McLTPII.9-GFP with the CW or PM marker, primarily at the cellular boundary; the exact localisation of McLTPII.9 could not be determined (**Figure 3**). Therefore, *N. benthamiana* leaf areas with co-expressing epidermis cells were plasmolysed. After plasmolysis, McLTPII.9-GFP was separated from the CW marker (**Figure 3A**), and co-localised with the PM marker (**Figure 3B**). Overlap of green and red fluorescence indicated that McLTPII.9 was localised to the plasma membrane, consistent with a previous transmembrane domain prediction (**Supplementary Figure S1B**).

### 3.3 Overexpression of *McLTPII.9* increases peltate glandular trichome density in *M. piperita*

*M. piperita* (peppermint) is a Mentha plant with similar glandular trichome structure and volatile oil to *M. canadensis*. Plant transformation in *M. piperita* is easier than in *M. canadensis*. To explore the biological function of *McLTPII.9* in mint, transgenic peppermint (*M. piperita*) lines overexpressing *McLTPII.9* were generated. Exogenous *McLTPII.9* expression was evaluated in the transgenic lines by genomic PCR and RT-qPCR (**Supplementary Figures S2A–C**). The overexpression vector had a GFP tag, which was fused to McLTPII.9. The transgenic lines showed clear signals of the GFP fragment (**Supplementary Figure S2A**) and McLTPII.9-GFP fragment (**Supplementary Figure S2B**), whereas the WT line did not. The expression levels of *McLTPII.9* were higher in the transgenic lines (OE1 and OE2) than in WT (**Supplementary Figure S2C**), and the expression in OE1 was twice as much as that in OE2. Moreover, confocal fluorescence imaging of OE1 leaves revealed that McLTPII.9-GFP was expressed in leaf cells, particularly in peltate glandular trichomes (**Supplementary Figure S2D**).

**Figure 4:**
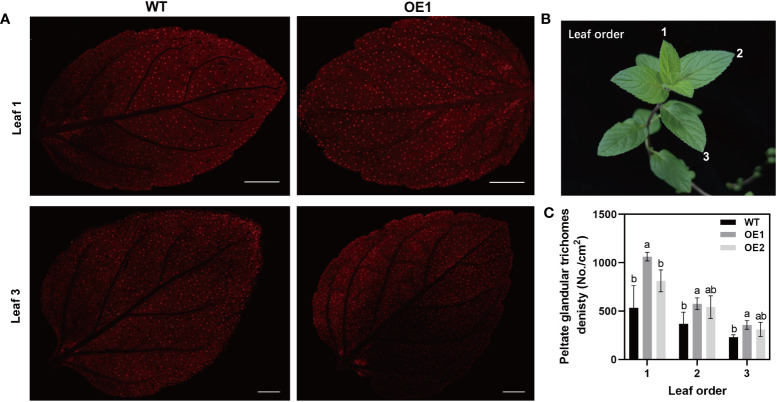
Peltate glandular trichome density in *McLTPII.9*-overexpressing *M. piperita* leaves. **(A)** Peltate glandular trichomes on the abaxial side of leaves from wild-type and McLTPII.9-overexpressing peppermint (OE1 and OE2). Bars, 2 mm. **(B)** Diagram of leaf position for observing. **(C)** Peltate glandular trichome densities in WT, OE1, and OE2 mint. Data are means ± standard deviations (SD) of three biological replicates. Different letters above each bar indicate significant differences (*p* < 0.05, Duncan’s multiple range test).

The peltate glandular trichome is important for the synthesis, secretion, and storage of volatile oil (Mahmoud et al., 2021). Under scanning electron microscopy (SEM), we observed the peltate glandular trichomes on abaxial side of WT and OE1 leaves (**Supplementary Figure S3**). The results showed that the density of peltate glandular trichome was increased in OE1 compared with that in WT. For accurate statistical analysis of peltate glandular trichomes, stereo fluorescence microscopy was used to count the number of peltate glandular trichomes on abaxial side of intact leaves. The peltate glandular trichome density in OE1 plants was significantly increased compared with density in the WT (**Figure 4**). The densities of the first, second, and third leaves in OE1 were 98%, 56%, and 58% greater than in the WT, respectively (**Figure 4C**). The difference between OE2 and WT was not significantly, but the mean density of the peltate glandular trichome were also increased compared with that of WT. These results may be related to the *McLTPII.9* expression levels in different transgenic lines. Furthermore, the peltate glandular trichome density decreased with leaf growth (**Figure 4**). Therefore, overexpression of *McLTPII.9* increased the peltate glandular trichome density in *M. piperita*.

### 3.4 Overexpression of *McLTPII.9* influences volatile oil content and composition in *M. piperita*

**Figure 5:**
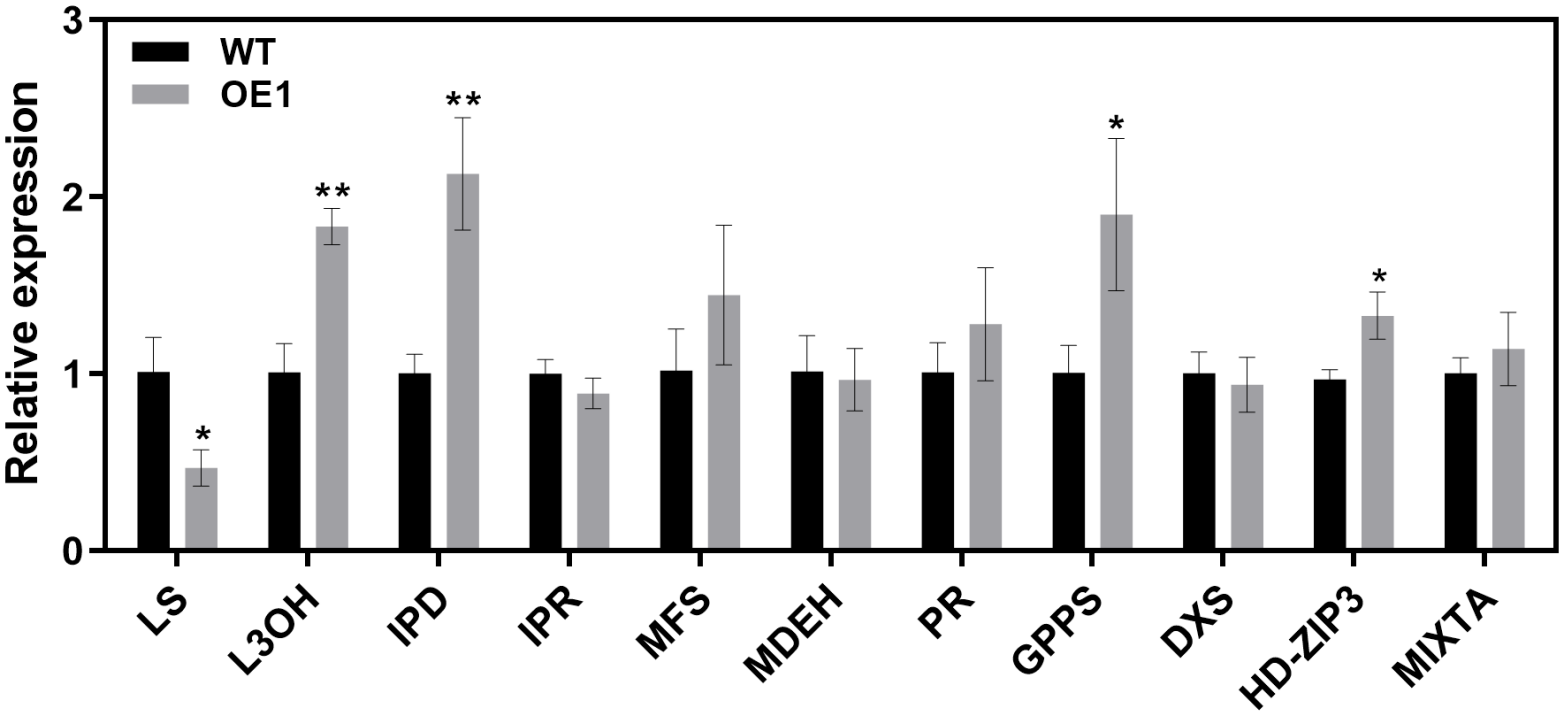
Expression levels of volatile oil synthesis and glandular trichome development-related genes in wild-type and *McLTPII.9*-overexpressing peppermint. Data are means ± SD of three biological replicates of three plants each. *p < 0.05; **p < 0.01, Student’s *t*-test. *MpActin1* was used as an internal control. Genes involved in volatile oil synthesis include *LS*, limonene synthase; *L3OH*, limonene-3-hydroxylase; *GPPS*, geranyl diphosphate synthase; *IPD*, transisopiperitenol dehydrogenase; *IPR*, isopiperitenone reductase; *PR*, pulegone reductase; *MDEH*, menthone dehydrogenase; *MFS*, menthofuran synthase; and *DXS*, 1-deoxy-D-xylulose-5-phosphate synthase. MIXTA is an R2R3-MYB transcription factor and HD-ZIP3 is an HD-ZIP IV transcription factor.

The peltate glandular trichomes in mint are rich in monoterpenoid volatile oils (Rios-Estepa et al., 2008). Our findings thus far suggested that *McLTPII.9* was associated with peltate glandular trichome density. To characterise *McLTPII.9* function, the extracted volatile compounds of *McLTPII.9*-overexpressing and WT plants were analysed by headspace solid-phase microextraction coupled with gas chromatography/mass spectrometry. More than 30 volatile compounds were identified in WT and OE lines using headspace solid-phase microextraction (**Table 1**). Based on their peak areas (**Supplementary Figure S4**), monoterpenoids were the most abundant, followed by sesquiterpenoids. Compared with the WT, most of the compounds in OE lines exhibited varying degrees of alteration. Eight monoterpenoids were significantly increased in OE1, and three were significantly increased in OE2; whereas four terpenoids were significantly decreased in both OE lines, including D-limonene, menthol, menthyl acetate, and α-Copaene. The total volatile compound content was greater in OE lines than in the WT. This finding suggested that McLTPII.9 influences volatile oil biosynthesis in peppermint. Next, we analysed several key enzyme genes and transcription factors involved in the synthesis of volatile oil and development of glandular trichomes (Rahimi et al., 2017; Qi et al., 2022). Compared with WT, the expression levels of limonene-3-hydroxylase (*L3OH*), transisopiperitenol dehydrogenase (*IPD*), geranyl diphosphate synthase (*GPPS*), and *HD-ZIP3* were significantly increased in *McLTPII.9*-overexpressing plant, whereas the expression level of limonene synthase (*LS*) was significantly decreased. However, the expression levels of isopiperitenone reductase (*IPR*), menthone dehydrogenase (*MDEH*), 1-deoxy-D-xylulose-5-phosphate synthase (*DXS*), pulegone reductase (*PR*), and *MIXTA* exhibited minimal changes (**Figure 5**). These results suggested that McLTPII.9-mediated changes in volatile oil and peltate glandular trichomes were accompanied by alterations in the expression patterns of related genes.

**Table 1:** Volatile organic compound composition in wild-type and transgenic peppermint plants, determined using headspace solid-phase microextraction.

No.	Retention time (min)	WT Peak area	(% to WT)	OE1 Peak area	(% to WT)	OE2 Peak area	(% to WT)	Compounds	Classification
1	6.42	574,440,219	100	1,114,704,801	194**	805,319,447	140*	α-Pinene	Monoterpenoid
2	7.22	706,430,938	100	1,209,576,991	171**	922,111,172	131	Sabinene	Monoterpenoid
3	7.34	1,047,765,600	100	1,789,364,938	171*	1,381,344,745	132	β-Pinene	Monoterpenoid
4	7.52	439,781,650	100	837,417,269	190**	628,335,378	134	α-Myrcene	Monoterpenoid
5	7.66	220,083,816	100	370,237,458	168**	283,739,738	129*	3-Octanol	Monoterpenoid
6	8.63	2,531,911,180	100	not detected	0**	not detected	0**	D-Limonene	Monoterpenoid
7	8.72	6,569,711,182	100	11,074,021,539	169**	9,927,417,362	109	Eucalyptol	Monoterpenoid
8	9.08	75,998,892	100	250,624,537	330**	120,240,908	158	α-Ocimene	Monoterpenoid
9	9.88	2,565,818,769	100	2,622,615,019	102	2,471,917,942	96	Sabinene hydrate	Monoterpenoid
10	10.58	100,342,117	100	120,497,768	120	108,272,637	108	Terpinolene	Monoterpenoid
11	11.01	424,348,083	100	492,725,139	116*	436,411,333	103	Linalool	Monoterpenoid
12	13.25	121,867,577	100	94,782,544	78	73,198,184	60	cis-Sabinol	Monoterpenoid
13	14.01	29,700,299,025	100	32,737,917,508	110	30,726,636,724	103	Menthone	Monoterpenoid
14	14.43	15,219,624,933	100	14,299,378,999	94	16,368,583,413	108	Menthofuran	Monoterpenoid
15	14.87	13,445,682,868	100	10,513,453,698	78**	10,675,188,848	79*	Menthol	Monoterpenoid
16	15.2	104,046,179	100	77,191,389	74	68,141,218	65	Isopulegol	Monoterpenoid
17	15.46	710,396,695	100	585,891,427	82	616,006,265	87	α-Terpineol	Monoterpenoid
18	16.91	6,946,731,712	100	7,285,184,732	105	8,475,501,033	122*	Pulegone	Monoterpenoid
19	17.28	585,671,948	100	578,363,236	99	491,572,241	84	Piperitone	Monoterpenoid
20	18.05	541,012,639	100	232,952,046	43**	288,867,721	53*	Menthyl acetate	Monoterpenoid
21	19.24	478,591,970	100	400,211,273	84	554,642,286	116	Menthofurolactone	Monoterpenoid
22	19.77	182,714,850	100	176,581,928	97	157,056,129	86	α-Bourbonene	Sesquiterpenoid
23	19.83	268,220,551	100	256,760,550	96	258,452,991	96	β-Elemene	Sesquiterpenoid
24	20.3	3,596,825,778	100	3,335,583,618	93	3,331,217,166	93	Caryophyllene	Sesquiterpenoid
25	20.41	141,426,619	100	104,938,026	74**	95,338,474	67**	α-Copaene	Sesquiterpenoid
26	20.63	860,270,978	100	777,241,892	90	798,937,220	93	β-Farnesene	Sesquiterpenoid
27	20.76	209,673,893	100	195,516,350	93	194,080,297	93	Humulene	Sesquiterpenoid
28	21.1	3,677,164,756	100	3,619,929,260	98	3,577,287,861	97	Germacrene D	Sesquiterpenoid
29	21.28	277,327,403	100	272,469,748	98	271,531,631	98	Elixene	Sesquiterpenoid
30	21.54	432,402,022	100	326,652,820	76	407,839,962	94	Tau-Cadinol acetate	Sesquiterpenoid
31	22.46	218,600,175	100	178,485,356	82	170,960,908	78	Caryophyllene oxide	Sesquiterpenoid
	others	606,637,717		595,332,946		800,878,361			
	Total area	93,581,822,733		97,087,222,649		95,487,029,592			

Data are mean volatile compound peak areas from three biological replicates, each containing 0.4 g dry leaves. Asterisks indicate significant differences from the WT (*p < 0.05; **p < 0.01, Student’s t-test).

## 4 Discussion

nsLTPs are multifunctional proteins essential for plant development, seed germination, cell signal transduction, and other biological processes (Sarowar et al., 2009; Chae et al., 2010; Huang et al., 2013; Cotta et al., 2014; Edstam and Edqvist, 2014; Zaidi et al., 2020; Chen et al., 2022). They transfer various hydrophobic molecules and are involved in the synthesis of lipid barrier polymers, such as cuticular waxes, suberin, and sporopollenin (Lee et al., 2009; Chen et al., 2011; Kim et al., 2012; Edstam et al., 2013; Song et al., 2020). In this study, we isolated an *nsLTP* gene belonging to the LTPII family from *M. canadensis*; we named it *McLTPII.9* based on the results of homology alignment (**Figure 1**). *McLTPII.9* was highly expressed in stems, leaves, and particularly young leaves of *M. canadensis* (**Figure 2**). We tried to transform promoter_McLTPII.9_::GUS plasmid to peppermint, unfortunately, no transgenic mint was obtained so far. So, we transformed recombinant plasmid into *N. tabacum* to verify the expression pattern. GUS signals driven by the *McLTPII.9* native promoter were detected in epidermal cells and long and short glandular trichomes of transgenic tobacco, suggesting that it is probably involved in glandular trichome development or metabolite secretion. nsLTPs, which constitute up to 32% of the cDNA library in mint glandular trichomes, may be linked to intracellular transport and secretion of volatile oils (Lange and Croteau, 1999; Tissier et al., 2017). In plants, the transport of volatile compounds requires carrier proteins (Widhalm et al., 2015), such as ABC and nsLTP. For example, *Petunia hybrida* PhABCG1 mediates the plasma membrane transport of benzenoid volatile compounds in petunia flowers (Adebesin et al., 2017). NtPDR1, a plasma membrane ABC transporter in tobacco, is involved in terpene transport (Crouzet et al., 2013). Together with *Artemisia annua* AaPDR2, AaLTP3 enhances the accumulation of dihydroartemisinic acid in apoplasts of *N. benthamiana* leaves (Wang et al., 2016). However, the biological roles and transport mechanisms of nsLTPs remain to be explored.

To successfully fulfil their roles in transport, nsLTPs must be targeted to the appropriate subcellular compartments. For example, AtLTP2 in the cell wall maintains cuticle-cell wall interface integrity to control etiolated hypocotyl permeability (Jacq et al., 2017). AtDIR1, a lipid transfer protein, is localised to the endoplasmic reticulum and cell periphery, where it mediates long-distance signalling during systemic acquired resistance (Champigny et al., 2011). AtLTPI-4, which is localised to the plasma membrane, functions in the delivery of long-chain and very long-chain fatty acids into the extracellular space for suberin assembly (Deeken et al., 2016). In this study, McLTPII.9 was localised to the plasma membrane (**Figure 3**), possibly in relation to the presence of a transmembrane signal peptide. *McLTPII.9* was expressed in glandular trichomes, the primary sites for the synthesis and storage of secondary metabolites. Therefore, the subcellular localisation of McLTPII.9 suggested that it might be involved in volatile metabolite transport in mint.

Some studies have indicated that nsLTPs mediate the transport of metabolites secreted by glandular trichomes. *NtLTP1* is implicated in long glandular trichome lipid secretion in tobacco; its overexpression in transgenic orange mint led to increased monoterpene emission (Choi et al., 2012). Overexpression of *AaLTP3* and *AaLTP4* in transgenic *A. annua* increased the sesquiterpene lactone content, compared with the WT (Adhikari et al., 2019). Additionally, overexpression of *BraLTP2* in *B. napus* increased the trichome number and altered secondary metabolite accumulation (Tian et al., 2018).

Using *Agrobacterium*-mediated transformation (Niu et al., 2000), we generated transgenic peppermints overexpressing *McLTPII.9*. The gene expression of OE1 was higher than that of OE2. The peltate glandular trichome densities on the first, second, and third leaf positions were significantly greater in OE1 than in WT (**Figure 4**). In OE2, peltate glandular trichome density were also increased compared with that in WT. These findings suggest that *McLTPII.9* is associated with the development of glandular trichomes. In our study, we examined the expression levels of some transcript factors related to mint glandular trichome development, such as *MpHD-ZIP3* and *MpMIXTA*, which were homologous with *McHD-ZIP3* and *McMIXTA*, respectively (**Figure 5**). The McHD-ZIP3 belongs to HD-ZIP IV subfamily, overexpression of McHD-ZIP3 in *N. tabacum* led to increased trichome number (Chen et al., 2020). McMIXTA formed a complex with trichome development-related McHD-ZIP3, and overexpression of McMIXTA in *M. piperita* led to increased peltate trichome density on the abaxial surface of leaves (Qi et al., 2022). In plants, many HD-ZIP IV transcription factors are important for glandular trichome initiation, such as AaHD1, AaHD8 and Wooly (Chalvin et al., 2019). AaHD8 directly and positively regulates the expression of AaHD1 in promoting trichome initiation. The transcriptional activity of AaHD8 was enhanced by interacting with AaMIXTA1 (Shi et al., 2018; Yan et al., 2018). In our results, the glandular trichome density was altered in the *McLTPII.9*-overexpressing plants as well as the expression of genes for transcription factors that were shown to be involved in trichome development in other plants (**Figure 5**). These results suggested that McLTPII.9 played a positive role in peltate glandular trichome development.

The accumulation of plant metabolites is associated with secretory glandular trichome density. A greater number of secretory glandular trichomes enhances the accumulation of secondary metabolites (Lange et al., 2000; Gao et al., 2018; Zhou et al., 2020; Gong et al., 2021; Guan et al., 2022). In the present study, overexpression of *McLTPII.9* led to an increase in volatile oil content, while changing the volatile oil composition (**Table 1**). The expression of genes in the terpene biosynthesis pathway affects volatile oil composition and content in mint (Krasnyanski et al., 1999; McConkey et al., 2000; Li C, et al., 2020). Overexpression of *MpLS* in peppermint resulted in high levels of menthone, menthofuran, and pulegone, as well as a low level of menthol, relative to WT (Krasnyanski et al., 1999). Inhibition of *MsLS* expression in spearmint led to reductions in limonene and carvone (Li C, et al., 2020 levels of key genes in the mint monoterpene synthesis pathway). Therefore, we examined the expression levels of key genes in the mint monoterpene synthesis pathway (Davis et al., 2005; Dolzhenko et al., 2010; Rahimi et al., 2017). Compared with WT, the expression level of *MpLS* in *McLTPII.9*-overexpressing peppermint was decreased, whereas the expression levels of *MpGPPS*, *MpL3OH*, and *MpIPD* were significantly increased (**Figure 5**). Changes in the expression levels of these genes corresponded to changes in volatile oil composition in transgenic peppermint. The content of limonene was lower in transgenic mint OE1 than in WT, and the peak area of limonene was not distinct from eucalyptol in the OE1 chromatogram (**Supplementary Figure S4**, **Table 1**). However, the α-pinene, β-pinene, and α-myrcene contents were increased in OE1. These results may be related to the increased expression levels of limonene downstream genes, such as *MpL3OH* and *MpIPD*, which promoted limonene consumption, or to the accumulation of by-products (e.g., α-pinene, β-pinene, and α-myrcene). Additionally, there were increases in the contents of some monoterpenoids derived from geranyl diphosphate (GPP), such as eucalyptol and linalool (**Table 1**). The increased expression of *MpGPPS* and decreased expression of *MpLS* in transgenic peppermint may explain the accumulation of GPP and GPP-derived products.

Although the menthol content was significantly lower in *McLTPII.9*-overexpressing plant than in WT, the menthone content was higher in *McLTPII.9*-overexpressing plant. Pulegone reductase (PR) catalyses the conversion of pulegone to menthone. The expression of *MpPR* in transgenic plant was slightly higher than in WT, presumably affecting menthone accumulation. Some studies suggested that the menthone was transported out of, and subsequently re-enter into, secretory cells during menthol synthesis (McConkey et al., 2000; Turner et al., 2012; Schuurink and Tissier, 2020). The reduced menthol may have been related to this reason. Overexpression of *McLTPII.9* may affect menthone transport and accumulation, thus influencing menthol synthesis. The transport function of McLTPII.9 needs further investigation.

## 5 Conclusion

We cloned and characterised the lipid transfer protein gene *McLTPII.9* from *M. canadensis*. McLTPII.9 was localised to the plasma membrane and was expressed in most tissues of *M. canadensis*. In *McLTPII.9-*overexpressing transgenic peppermint, the peltate glandular trichome density and volatile oil content were increased, compared with WT. Additionally, the volatile oil composition was altered. Expression analysis of monoterpene biosynthesis-related genes and developmental transcription factors implicated *McLTPII.9* in the positive regulation of glandular trichome density and monoterpene metabolism. Our results provide a theoretical basis for mint breeding and terpene metabolic engineering.

## Data availability statement

The original contributions presented in the study are included in the article/**Supplementary Material**. Further inquiries can be directed to the corresponding authors.

## Author contributions

LL and CL designed the experiments. QC and LL performed the experiments, analysed the data, and wrote the manuscript. YB, DL and QL provided support for plant growth. XQ helped in bioinformatics analysis. HF, XY and ZC provided the materials and technical support. All authors contributed to the article and approved the submitted version.

## References

[B1] Adebesin F. Widhalm J. R. Boachon B. Lefèvre F. Pierman B. Lynch J. H. (2017). Emission of volatile organic compounds from petunia flowers is facilitated by an ABC transporter. Science 356, 1386–1388. doi: 10.1126/science.aan0826 28663500

[B2] Adhikari P. B. Han J. Y. Ahn C. H. Choi Y. E. (2019). Lipid transfer proteins (AaLTP3 and AaLTP4) are involved in sesquiterpene lactone secretion from glandular trichomes in *Artemisia annua* . Plant Cell Physiol. 60, 2826–2836. doi: 10.1093/pcp/pcz171 31504880

[B3] Boutrot F. Chantret N. Gautier M. F. (2008). Genome-wide analysis of the rice and arabidopsis non-specific lipid transfer protein (nsLTP) gene families and identification of wheat nsLTP genes by EST data mining. BMC Genomics 9, 86. doi: 10.1186/1471-2164-9-86 18291034 PMC2277411

[B4] Cameron K. D. Teece M. A. Smart L. B. (2006). Increased accumulation of cuticular wax and expression of lipid transfer protein in response to periodic drying events in leaves of tree tobacco. Plant Physiol. 140, 176–183. doi: 10.1104/pp.105.069724 16361524 PMC1326042

[B5] Chae K. Gonong B. J. Kim S. C. Kieslich C. A. Morikis D. Balasubramanian S.. (2010). A multifaceted study of stigma/style cysteine-rich adhesin (SCA)-like arabidopsis lipid transfer proteins (LTPs) suggests diversified roles for these LTPs in plant growth and reproduction. J. Exp. Bot. 61, 4277–4290. doi: 10.1093/jxb/erq228 20667964 PMC2955742

[B6] Chalvin C. Drevensek S. Dron M. Bendahmane A. Boualem A. (2019). Genetic control of glandular trichome development. Trends Plant Sci. 25, 477–487. doi: 10.1016/j.tplants.2019.12.025 31983619

[B7] Champigny M. J. Isaacs M. Carella P. Faubert J. Fobert P. R. Cameron R. K. (2013). Long distance movement of DIR1 and investigation of the role of DIR1-like during systemic acquired resistance in arabidopsis. Front. Plant Sci. 4. doi: 10.3389/fpls.2013.00230 PMC3701462 23847635

[B8] Champigny M. J. Shearer H. Mohammad A. Haines K. Neumann M. Thilmony R.. (2011). Localization of DIR1 at the tissue, cellular and subcellular levels during systemic acquired resistance in arabidopsis using DIR1:GUS and DIR1:EGFP reporters. BMC Plant Biol. 11, 125. doi: 10.1186/1471-2229-11-125 21896186 PMC3180652

[B9] Chen C. Chen G. Hao X. Cao B. Chen Q. Liu S.. (2011). CaMF2, an anther-specific lipid transfer protein (LTP) gene, affects pollen development in *Capsicum annuum* l. Plant Sci. 181, 439–448. doi: 10.1016/j.plantsci.2011.07.003 21889050

[B10] Chen L. Ji C. Zhou D. Gou X. Tang J. Jiang Y.. (2022). OsLTP47 may function in a lipid transfer relay essential for pollen wall development in rice. J. Genet. Genomics 49, 481–491. doi: 10.1016/j.jgg.2022.03.003 35331929

[B11] Chen Z. Qi X. Fang H. Yu X. Li L. Liang C. (2020). Cloning of McHD-Zip3 gene of *Mentha canadensis* and function analysis on its regulatory of development of glandular trichomes. J. Plant Resour.& Environ. 29, 1–10. doi: 10.3969/j.issn.1674-7895.2020.0301

[B12] Choi Y. E. Lim S. Kim H. J. Han J. Y. Lee M. H. Yang Y.. (2012). Tobacco NtLTP1, a glandular-specific lipid transfer protein, is required for lipid secretion from glandular trichomes. Plant J. 70, 480–491. doi: 10.1111/j.1365-313X.2011.04886.x 22171964

[B13] Cotta M. G. Barros L. M. de Almeida J. D. de Lamotte F. Barbosa E. A. Vieira N. G.. (2014). Lipid transfer proteins in coffee: isolation of coffea orthologs, coffea arabica homeologs, expression during coffee fruit development and promoter analysis in transgenic tobacco plants. Plant Mol. Biol. 85, 11–31. doi: 10.1007/s11103-013-0166-5 24469961

[B14] Crouzet J. Roland J. Peeters E. Trombik T. Ducos E. Nader J.. (2013). NtPDR1, a plasma membrane ABC transporter from nicotiana tabacum, is involved in diterpene transport. Plant Mol. Biol. 82, 181–192. doi: 10.1007/s11103-013-0053-0 23564360

[B15] Davis E. M. Ringer K. L. McConkey M. E. Croteau R. (2005). Monoterpene metabolism. cloning, expression, and characterization of menthone reductases from peppermint. Plant Physiol. 137, 873–881. doi: 10.1104/pp.104.053306 15728344 PMC1065388

[B16] Deeken R. Saupe S. Klinkenberg J. Riedel M. Leide J. Hedrich R.. (2016). The nonspecific lipid transfer protein AtLTPI-4 is involved in suberin formation of arabidopsis thaliana crown galls. Plant Physiol. 172, 1911–1927. doi: 10.1104/pp.16.01486 27688623 PMC5100791

[B17] Diz M. S. Carvalho A. O. Ribeiro S. F. Da Cunha M. Beltramini L. Rodrigues R.. (2011). Characterisation, immunolocalisation and antifungal activity of a lipid transfer protein from chili pepper (*Capsicum annuum*) seeds with novel α-amylase inhibitory properties. Physiol. Plant 142, 233–246. doi: 10.1111/j.1399-3054.2011.01464.x 21382036

[B18] Dolzhenko Y. Bertea C. M. Occhipinti A. Bossi S. Maffei M. E. (2010). UV-B modulates the interplay between terpenoids and flavonoids in peppermint (*Mentha* ×*piperita* l.). J. Photochem. Photobiol. B. 100, 67–75. doi: 10.1016/j.jphotobiol.2010.05.003 20627615

[B19] Do Nascimento L. D. de Moraes A. A. B. da Costa K. S. Galúcio J. M. P. Taube P. S. Costa C. M. L.. (2020). Bioactive natural compounds and antioxidant activity of essential oils from spice plants: new findings and potential applications. Biomolecules 10, 988–1037. doi: 10.3390/biom10070988 32630297 PMC7407208

[B20] Eberl F. Gershenzon J. (2017). Releasing plant volatiles, as simple as ABC. Science 356, 1334–1335. doi: 10.1126/science.aan8291 28663457

[B21] Edqvist J. Blomqvist K. Nieuwland J. Salminen T. A. (2018). Plant lipid transfer proteins: are we finally closing in on the roles of these enigmatic proteins? J. Lipid Res. 59, 1374–1382. doi: 10.1194/jlr.R083139 29555656 PMC6071764

[B22] Edstam M. M. Blomqvist K. Eklöf A. Wennergren U. Edqvist J. (2013). Coexpression patterns indicate that GPI-anchored non-specific lipid transfer proteins are involved in accumulation of cuticular wax, suberin and sporopollenin. Plant Mol. Biol. 83, 625–649. doi: 10.1007/s11103-013-0113-5 23893219

[B23] Edstam M. M. Edqvist J. (2014). Involvement of GPI-anchored lipid transfer proteins in the development of seed coats and pollen in *Arabidopsis thaliana* . Physiol. Plant 152, 32–42. doi: 10.1111/ppl.12156 24460633

[B24] Edstam M. M. Laurila M. Höglund A. Raman A. Dahlström K. M. Salminen T. A.. (2014). Characterization of the GPI-anchored lipid transfer proteins in the moss physcomitrella patens. Plant Physiol. Biochem. 75, 55–69. doi: 10.1016/j.plaphy.2013.12.001 24374350

[B25] Edstam M. M. Viitanen L. Salminen T. A. Edqvist J. (2011). Evolutionary history of the non-specific lipid transfer proteins. Mol. Plant 4, 947–964. doi: 10.1093/mp/ssr019 21486996

[B26] Gani U. Vishwakarma R. A. Misra P. (2021). Membrane transporters: the key drivers of transport of secondary metabolites in plants. Plant Cell Rep. 40, 1–18. doi: 10.1007/s00299-020-02599-9 32959124

[B27] Gao Q. M. Kane N. C. Hulke B. S. Reinert S. Pogoda C. S. Tittes S.. (2018). Genetic architecture of capitate glandular trichome density in florets of domesticated sunflower (*Helianthus annuus* l.). Front. Plant Sci. 8. doi: 10.3389/fpls.2017.02227 PMC5767279 29375602

[B28] Gershenzon J. McConkey M. E. Croteau R. B. (2000). Regulation of monoterpene accumulation in leaves of peppermint. Plant Physiol. 122, 205–214. doi: 10.1104/pp.122.1.205 10631264 PMC58859

[B29] Gong Z. Luo Y. Zhang W. Jian W. Zhang L. Gao X.. (2021). A SlMYB75-centred transcriptional cascade regulates trichome formation and sesquiterpene accumulation in tomato. J. Exp. Bot. 72, 3806–3820. doi: 10.1093/jxb/erab086 33619530

[B30] Guan Y. Chen S. Chen F. Chen F. Jiang Y. (2022). Exploring the relationship between trichome and terpene chemistry in chrysanthemum. Plants (Basel). 11, 1410. doi: 10.3390/plants11111410 35684184 PMC9182802

[B31] Horsch R. B. Rogers S. G. Fraley R. T. (1985). Transgenic plants. Cold Spring Harb. Symp. Quant. Biol. 50, 433–437. doi: 10.1101/sqb.1985.050.01.054 3868487

[B32] Hua B. Chang J. Xu Z. Han X. Xu M. Yang M.. (2021). HOMEODOMAIN PROTEIN8 mediates jasmonate-triggered trichome elongation in tomato. New Phytol. 230, 1063–1077. doi: 10.1111/nph.17216 33474772

[B33] Huang M. D. Chen T. L. Huang A. H. (2013). Abundant type III lipid transfer proteins in arabidopsis tapetum are secreted to the locule and become a constituent of the pollen exine. Plant Physiol. 163, 1218–1229. doi: 10.1104/pp.113.225706 24096413 PMC3813645

[B34] Huchelmann A. Boutry M. Hachez C. (2017). Plant glandular trichomes: natural cell factories of high biotechnological interest. Plant Physiol. 175, 6–22. doi: 10.1104/pp.17.00727 28724619 PMC5580781

[B35] Hwang H. S. Adhikari P. B. Jo H. J. Han J. Y. Choi Y. E. (2020). Enhanced monoterpene emission in transgenic orange mint (*Mentha × piperita* f. citrata) overexpressing a tobacco lipid transfer protein (NtLTP1). Planta 252, 44. doi: 10.1007/s00425-020-03447-6 32876749

[B36] Jacq A. Pernot C. Martinez Y. Domergue F. Payré B. Jamet E.. (2017). The *Arabidopsis* lipid transfer protein 2 (AtLTP2) is involved in cuticle-cell wall interface integrity and in etiolated hypocotyl permeability. Front. Plant Sci. 8. doi: 10.3389/fpls.2017.00263 PMC5326792 28289427

[B37] Jefferson R. A. Kavanagh T. A. Bevan M. W. (1987). GUS fusions: beta-glucuronidase as a sensitive and versatile gene fusion marker in higher plants. EMBO J. 6, 3901–3907. doi: 10.1002/j.1460-2075.1987.tb02730.x 3327686 PMC553867

[B38] Jin J. Panicker D. Wang Q. Kim M. J. Liu J. Yin J. L.. (2014). Next generation sequencing unravels the biosynthetic ability of spearmint (Mentha spicata) peltate glandular trichomes through comparative transcriptomics. BMC Plant Biol. 14, 292. doi: 10.1186/s12870-014-0292-5 25367433 PMC4232691

[B39] Karpiński T. M. (2020). Essential oils of lamiaceae family plants as antifungals. Biomolecules 10, 103. doi: 10.3390/biom10010103 31936168 PMC7023020

[B40] Kelley L. A. Mezulis S. Yates C. M. Wass M. N. Sternberg M. J. (2015). The Phyre2 web portal for protein modeling, prediction and analysis. Nat. Protoc. 10, 845–858. doi: 10.1038/nprot.2015.053 25950237 PMC5298202

[B41] Kim H. Lee S. B. Kim H. J. Min M. K. Hwang I. Suh M. C. (2012). Characterization of glycosylphosphatidylinositol-anchored lipid transfer protein 2 (LTPG2) and overlapping function between LTPG/LTPG1 and LTPG2 in cuticular wax export or accumulation in *Arabidopsis thaliana* . Plant Cell Physiol. 53, 1391–1403. doi: 10.1093/pcp/pcs083 22891199

[B42] Krasnyanski S. May R. A. Loskutov A. Ball T. M. Sink K. C. (1999). Transformation of the limonene synthase gene into peppermint (*Mentha piperita* l.) and preliminary studies on the essential oil profiles of single transgenic plants. Theor. Appl. Genet. 99, 676–682. doi: 10.1007/s001220051284 22665205

[B43] Lange B. M. Croteau R. (1999). Genetic engineering of essential oil production in mint. Curr. Opin. Plant Biol. 2, 139–144. doi: 10.1016/s1369-5266(99)80028-4 10322195

[B44] Lange B. M. Wildung M. R. Stauber E. J. Sanchez C. Pouchnik D. Croteau R. (2000). Probing essential oil biosynthesis and secretion by functional evaluation of expressed sequence tags from mint glandular trichomes. Proc. Natl. Acad. Sci. 97, 2934–2939. doi: 10.1073/pnas.97.6.2934 10717007 PMC16033

[B45] Lee S. B. Go Y. S. Bae H. J. Park J. H. Cho S. H. Cho H. J.. (2009). Disruption of glycosylphosphatidylinositol-anchored lipid transfer protein gene altered cuticular lipid composition, increased plastoglobules, and enhanced susceptibility to infection by the fungal pathogen *Alternaria brassicicola* . Plant Physiol. 150, 42–54. doi: 10.1104/pp.109.137745 19321705 PMC2675750

[B46] Li L. Li N. Fang H. Qi X. Zhou Y. (2020). Selection and validation of reference genes for normalisation of gene expression in *Glehnia littoralis* . Sci. Rep. 10, 7374. doi: 10.1038/s41598-020-63917-5 32355237 PMC7192926

[B47] Li C. Sarangapani S. Wang Q. Nadimuthu K. Sarojam R. (2020). Metabolic engineering of the native monoterpene pathway in spearmint for production of heterologous monoterpenes reveals complex metabolism and pathway interactions. Int. J. Mol. Sci. 21, 6164. doi: 10.3390/ijms21176164 32859057 PMC7504178

[B48] Li L. Wang F. Yan P. Jing W. Zhang C. Kudla J.. (2017). A phosphoinositide-specific phospholipase c pathway elicits stress-induced Ca2+ signals and confers salt tolerance to rice. New Phytol. 214, 1172–1187. doi: 10.1111/nph.14426 28157263

[B49] Liu W. Huang D. Liu K. Hu S. Yu J. Gao G.. (2010). Discovery, identification and comparative analysis of non-specific lipid transfer protein (nsLTP) family in solanaceae. Genom. Proteom. Bioinf. 8, 229–237. doi: 10.1016/S1672-0229(10)60024-1 PMC5054125 21382591

[B50] Liu R. Wang Y. Liang C. Zheng Z. Du X. Cui Z.. (2023). Morphology and mass spectrometry-based chemical profiling of peltate glandular trichomes on *Mentha haplocalyx* briq leaves. Food Res. Int. 164, 112323. doi: 10.1016/j.foodres.2022.112323 36737916

[B51] Liu F. Xiong X. Wu L. Fu D. Hayward A. Zeng X.. (2014). BraLTP1, a lipid transfer protein gene involved in epicuticular wax deposition, cell proliferation and flower development in brassica napus. PloS One 9, e110272. doi: 10.1371/journal.pone.0110272 25314222 PMC4196963

[B52] Liu F. Zhang X. Lu C. Zeng X. Li Y. Fu D.. (2015). Non-specific lipid transfer proteins in plants: presenting new advances and an integrated functional analysis. J. Exp. Bot. 66, 5663–5681. doi: 10.1093/jxb/erv313 26139823

[B53] Madni Z. K. Tripathi S. K. Salunke D. M. (2020). Structural insights into the lipid transfer mechanism of a non-specific lipid transfer protein. Plant J. 102, 340–352. doi: 10.1111/tpj.14627 31793077

[B54] Mahendran G. Rahman L. U. (2020). Ethnomedicinal, phytochemical and pharmacological updates on peppermint (*Mentha × piperita* l.)-a review. Phytother. Res. 34, 2088–2139. doi: 10.1002/ptr.6664 32173933

[B55] Mahmoud S. S. Maddock S. Adal A. M. (2021). Isoprenoid metabolism and engineering in glandular trichomes of *Lamiaceae* . Front. Plant Sci. 12. doi: 10.3389/fpls.2021.699157 PMC8326662 34349773

[B56] McConkey M. E. Gershenzon J. Croteau R. B. (2000). Developmental regulation of monoterpene biosynthesis in the glandular trichomes of peppermint. Plant Physiol. 122, 215–224. doi: 10.1104/pp.122.1.215 10631265 PMC58860

[B57] Missaoui K. Gonzalez-Klein Z. Pazos-Castro D. Hernandez-Ramirez G. Garrido-Arandia M. Brini F.. (2022). Plant non-specific lipid transfer proteins: an overview. Plant Physiol. Biochem. 171, 115–127. doi: 10.1016/j.plaphy.2021.12.026 34992048

[B58] Niu X. Li X. Veronese P. Bressan R. A. Weller S. C. Hasegawa P. M. (2000). Factors affecting *Agrobacterium tumefaciens*-mediated transformation of peppermint. Plant Cell Rep. 19, 304–310. doi: 10.1007/s002990050017 30754913

[B59] Niu X. Lin K. Hasegawa P. M. Bressan R. A. Weller S. C. (1998). Transgenic peppermint (*Mentha×piperita* l.) plants obtained by cocultivation with *Agrobacterium tumefaciens* . Plant Cell Rep. 17, 165–171. doi: 10.1007/s002990050372 30736494

[B60] Pagnussat L. Burbach C. Baluska F. de la Canal L. (2012). An extracellular lipid transfer protein is relocalized intracellularly during seed germination. J. Exp. Bot. 63, 6555–6563. doi: 10.1093/jxb/ers311 23162115

[B61] Qi X. Chen Z. Yu X. Li L. Bai Y. Fang H.. (2022). Characterisation of the *Mentha canadensis* R2R3-MYB transcription factor gene McMIXTA and its involvement in peltate glandular trichome development. BMC Plant Biol. 22, 219. doi: 10.1186/s12870-022-03614-9 35477355 PMC9047286

[B62] Qi X. Fang H. Yu X. Xu D. Li L. Liang C.. (2018). Transcriptome analysis of ja signal transduction, transcription factors, and monoterpene biosynthesis pathway in response to methyl jasmonate elicitation in *Mentha canadensis* l. Int. J. Mol. Sci. 19, 2364. doi: 10.3390/ijms19082364 30103476 PMC6121529

[B63] Rahimi Y. Taleei A. Ranjbar M. (2017). Changes in the expression of key genes involved in the biosynthesis of menthol and menthofuran in *Mentha piperita* l. under drought stress. Acta Physiol. Plant 39, 203. doi: 10.1007/s11738-017-2502-x

[B64] Rios-Estepa R. Turner G. W. Lee J. M. Croteau R. B. Lange B. M. (2008). A systems biology approach identifies the biochemical mechanisms regulating monoterpenoid essential oil composition in peppermint. Proc. Natl. Acad. Sci. 105, 2818–2823. doi: 10.1073/pnas.0712314105 18287058 PMC2268543

[B65] Salminen T. A. Eklund D. M. Joly V. Blomqvist K. Matton D. P. Edqvist J. (2018). Deciphering the evolution and development of the cuticle by studying lipid transfer proteins in mosses and liverworts. Plants(Basel) 7, 6. doi: 10.3390/plants7010006 29342939 PMC5874595

[B66] Sarowar S. Kim Y. J. Kim K. D. Hwang B. K. Ok S. H. Shin J. S. (2009). Overexpression of lipid transfer protein (LTP) genes enhances resistance to plant pathogens and LTP functions in long-distance systemic signaling in tobacco. Plant Cell Rep. 28, 419–427. doi: 10.1007/s00299-008-0653-3 19089429

[B67] Schuurink R. Tissier A. (2020). Glandular trichomes: micro-organs with model status? New Phytol. 225, 2251–2266. doi: 10.1111/nph.16283 31651036

[B68] Shi P. Fu X. Shen Q. Liu M. Pan Q. Tang Y.. (2018). The roles of AaMIXTA1 in regulating the initiation of glandular trichomes and cuticle biosynthesis in artemisia annua. New Phytol. 217, 261–276. doi: 10.1111/nph.14789 28940606

[B69] Song X. Li E. Song H. Du G. Li S. Zhu H.. (2020). Genome-wide identification and characterization of nonspecific lipid transfer protein (nsLTP) genes in *Arachis duranensis* . Genomics 112, 4332–4341. doi: 10.1016/j.ygeno.2020.07.034 32717318

[B70] Tapia G. Morales-Quintana L. Parra C. Berbel A. Alcorta M. (2013). Study of nsLTPs in lotus japonicus genome reveal a specific epidermal cell member (LjLTP10) regulated by drought stress in aerial organs with a putative role in cutin formation. Plant Mol. Biol. 82, 485–501. doi: 10.1007/s11103-013-0080-x 23733601

[B71] Tian N. Liu F. Wang P. Yan X. Gao H. Zeng X.. (2018). Overexpression of *BraLTP2*, a lipid transfer protein of *Brassica napus*, results in increased trichome density and altered concentration of secondary metabolites. Int. J. Mol. Sci. 19, 1733. doi: 10.3390/ijms19061733 29895724 PMC6032385

[B72] Tissier A. Morgan J. A. Dudareva N. (2017). Plant volatiles: going 'in' but not 'out' of trichome cavities. Trends Plant Sci. 22, 930–938. doi: 10.1016/j.tplants.2017.09.001 28958712

[B73] Turner G. W. Davis E. M. Croteau R. B. (2012). Immunocytochemical localization of short-chain family reductases involved in menthol biosynthesis in peppermint. Planta 235 (6), 1185–1195. doi: 10.1007/s00425-011-1567-9 22170164

[B74] Turner G. W. Gershenzon J. Croteau R. B. (2000). Distribution of peltate glandular trichomes on developing leaves of peppermint. Plant Physiol. 124, 655–664. doi: 10.1104/pp.124.2.655 11027715 PMC59171

[B75] Turner G. W. Gershenzon J. Nielson E. E. Froehlich J. E. Croteau R. (1999). Limonene synthase, the enzyme responsible for monoterpene biosynthesis in peppermint, is localized to leucoplasts of oil gland secretory cells. Plant Physiol. 120, 879–886. doi: 10.1104/pp.120.3.879 10398724 PMC59327

[B76] Wang B. Kashkooli A. B. Sallets A. Ting H. M. de Ruijter N. C. A. Olofsson L.. (2016). Transient production of artemisinin in nicotiana benthamiana is boosted by a specific lipid transfer protein from *A. annua* . Metab. Eng. 38, 159–169. doi: 10.1016/j.ymben.2016.07.004 27421621

[B77] Widhalm J. R. Jaini R. Morgan J. A. Dudareva N. (2015). Rethinking how volatiles are released from plant cells. Trends Plant Sci. 20 (9), 545–550. doi: 10.1016/j.tplants.2015.06.009 26189793

[B78] Wu Z. Tan B. Liu Y. Dunn J. Martorell Guerola P. Tortajada M.. (2019). Chemical composition and antioxidant properties of essential oils from peppermint, native spearmint and scotch spearmint. Molecules 24, 2825. doi: 10.3390/molecules24152825 31382468 PMC6696458

[B79] Yan T. Li L. Xie L. Chen M. Shen Q. Pan Q.. (2018). A novel HD-ZIP IV/MIXTA complex promotes glandular trichome initiation and cuticle development in artemisia annua. New Phytol. 218, 567–578. doi: 10.1111/nph.15005 29377155

[B80] Yu X. Chen Z. Li S. Qi X. Fang H. Bai Y.. (2022). A stable method for agrobacterium-mediated transformation of mentha piperita and mentha canadensis using internodal explants. In Vitro Cell DEV-PL. 58, 1038–1047. doi: 10.1007/s11627-022-10294-5

[B81] Yu X. Qi X. Li S. Fang H. Bai Y. Li L.. (2021). Transcriptome analysis of light-regulated monoterpenes biosynthesis in leaves of *Mentha canadensis* l. Plants (Basel). 10, 930. doi: 10.3390/plants10050930 34066919 PMC8148558

[B82] Zaidi M. A. O’Leary S. J. B. Gagnon C. Chabot D. Wu S. Hubbard K.. (2020). A triticale tapetal non-specific lipid transfer protein (nsLTP) is translocated to the pollen cell wall. Plant Cell Rep. 39, 1185–1197. doi: 10.1007/s00299-020-02556-6 32638075

[B83] Zhou Z. Tan H. Li Q. Li Q. Wang Y. Bu Q.. (2020). TRICHOME AND ARTEMISININ REGULATOR 2 positively regulates trichome development and artemisinin biosynthesis in *Artemisia annua* . New Phytol. 228, 932–945. doi: 10.1111/nph.16777 32589757

