# Supplementary material for: The non-specific lipid transfer protein McLTPII.9 of Mentha canadensis is involved in peltate glandular trichome density and volatile compound metabolism
Source: Front Plant Sci. 2023 May 31;14:1188922. doi: 10.3389/fpls.2023.1188922 (PMC10264783; doi:10.3389/fpls.2023.1188922)
Supplement: Supplementary file 1 [file DataSheet_1.docx]

***McLTPII.9* CDS**

ATGAACACCAAAGCAGCATCCCTAGCAATCCTGGCTCTAGCAATGCTGGTTCTCGCCGAAGTGGAAGTCGCCGGCGCCGTGACGTGCGACCCGGTGCAGCTCAGCCCGTGCGCCGCCGCGATAACCTCGTCGGGGAAGCCGAGCGCCGCCTGCTGTGCGAAGCTGAAGGAGCAGAGGCCATGTCTGTGTACGTACATAAAGAATCCCAATCTGCAGAAGTTCATCAATTCTCCTGGGGCCAAGAAAGTTTCCAGTGCTTGTCACATTCCATACCCTAAGTGCTGA

**McLTPII.9 Protein**

MNTKAASLAILALAMLVLAEVEVAGAVTCDPVQLSPCAAAITSSGKPSAACCAKLKEQRPCLCTYIKNPNLQKFINSPGAKKVSSACHIPYPKC

***McLTPII.9* 1313-bp promoter**

TGTCGCCATCTCAACAGTTGGTACATTAAGTATATCAAAGATGGGTAGTGGAGCATTACTCTGTACAACGATAGTTCGTATGAAGAATTTATACATGTGTTGGGGAAAACGAAAGAAAGAGTTTGAAGGTACAAATATCACGTAAAATGGAAAGGTATTGTTGGTTGGGAAAACGGAAAAGCAAGAGGAGGAAGAAAATTTTGGAATTTCCTTCCGTTTCGTTTACTATTTAATTAAAATGGAAAAATTTATAGTTAAATCGAAACATATGAAGAGATTTGCATCCAAACTTCACGATTGATACGTTAATTAATGTTCTAATTTATTGCATTATTTTCCTTTTACATTTACTAATTTATTGAACCATTTCTTAATACAAAATAAAAATGTGTATATTTATGATATGCTCCAAACTCCGATGTGAATAAATGTTTCGCACTTTTCTTGTAGTTAATGCAGCTAGCTAGCTAGCTACAAAAATAAATAAATAAATAAATAAATAATGACTTATAATTTTAGCATGCAGGAATTTTTCATAAAATATAATAAAATAAATAAAAATTACACCATTTATAATATGAATGATCCAAAGCAAACAATGTGGCATGTGTAGAAACAATTGAGTCATGATTCCACTTATAATTACCAATGTTCTAAAAGTCGTCAGTCGCTAGTCGAGCGGTGGCGAGGGGACTAACGCCTAGGCGGCTAGGCGGGAGCCTAAGCGGATTAGAAACATTGTGCTTATTTTTATTATTTTTACATTATGTCATCAGTTAGAAACTATATTACATATATTATTCATAATTTTATTACCAAATTCATGATAAAAATATATAATATGATGATTAATAGTCCAATTCAATAAATTTCATAGTATACAATAAATTTTTCTAACTTTCAACATAATATTAATATATATTGAATTCAATATTTGTAATCTAATTTAGCACTAAATTCGTCTAGCCATCCAAATCACGTATGCAAACAGCCTAGTTAACGTAAATATCGATTAAACATACCTAGAGACTAATTAAACTTTAATATGACCAATAGTTAGGATGTCGCTAAGTGCCTAGTCGAATTTAGCATTAGTCGGGTCGGTGAGGCGTCGCCTAACGCCTAGGCGGAGACTAGGGGGGCCTAGGCGGGGACTTTTAGAACACTGACAATTACAAATCTAGCACCAAAGCAATGTGAACAAGAGATCTCTTTCTTCTCTCTCCATCCCTCTATAAATTCAGCCACAAACCACTCAATTTCCTCACTCACACACACACACACACATAATATTCACATACGAACAAACGAAA
