# Supplementary material for: The non-specific lipid transfer protein McLTPII.9 of Mentha canadensis is involved in peltate glandular trichome density and volatile compound metabolism
Source: Front Plant Sci. 2023 May 31;14:1188922. doi: 10.3389/fpls.2023.1188922 (PMC10264783; doi:10.3389/fpls.2023.1188922)
Supplement: Supplementary file 2 [file DataSheet_2.docx]

Supplementary Material

The non-specific lipid transfer protein McLTPII.9 of *Mentha canadensis* is involved in peltate glandular trichome density and volatile compound metabolism

**Qiutong Chen, Li Li^*^, Xiwu Qi, Hailing Fang, Xu Yu, Yang Bai, Zequn Chen, Qun Liu, Dongmei Liu, Chengyuan Liang^*^**

*** Correspondence:** Chengyuan Liang:[liangcy618@cnbg.net](mailto:liangcy618@cnbg.net); Li Li:xinwenbanlili@163.com


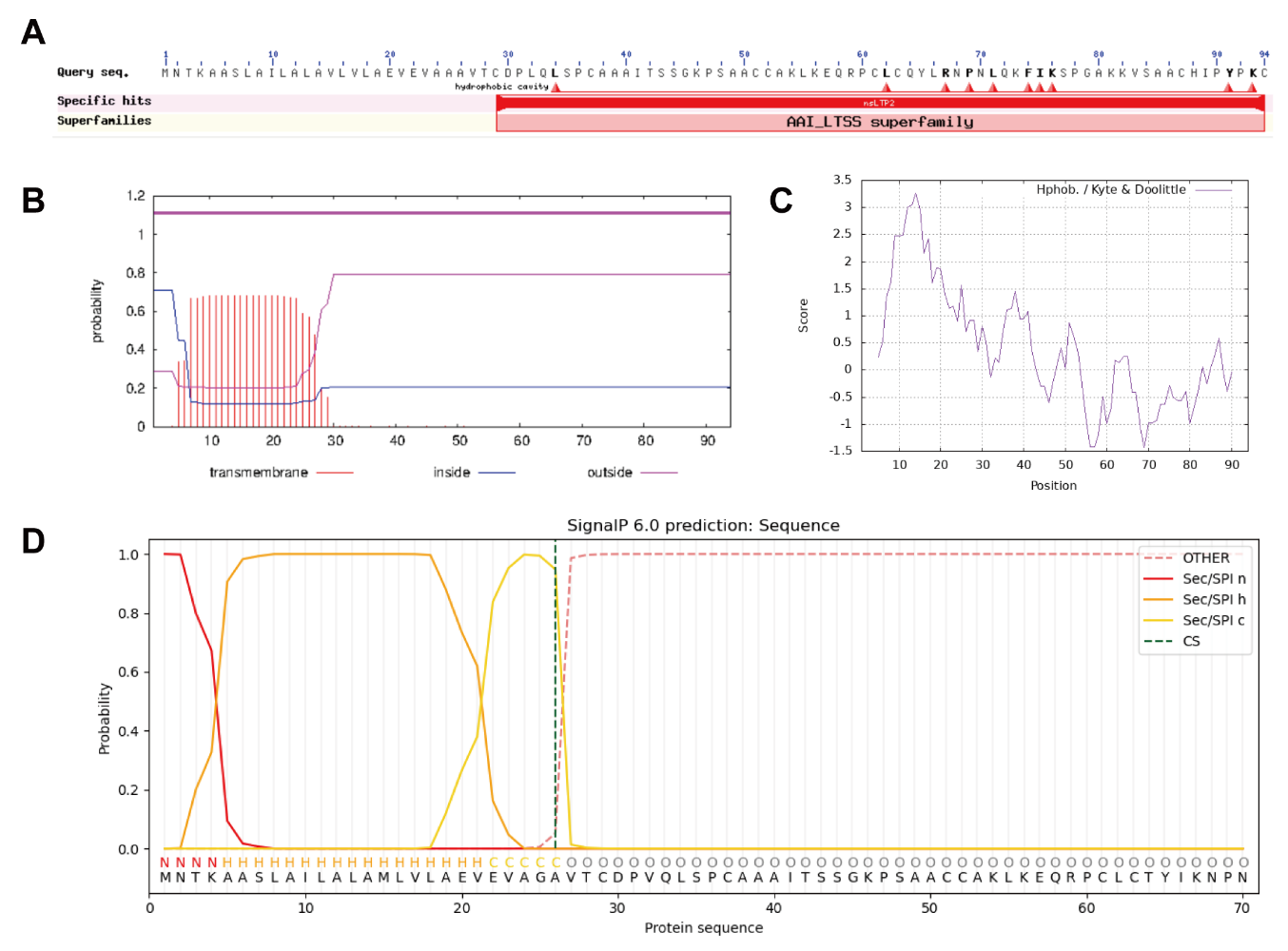


**Supplementary** **Figure S1. Bioinformatics features of McLTPII.9.**

(A) The National Centre for Biotechnology Information CD-Search tool was used to predict conserved domains in McLTPII.9, revealing that the protein belonged to the nsLTPII class. (B) Predicted transmembrane domains in McLTPII.9, determined using TMHMM 2.0. y-axis, possibility; red line, transmembrane domain; blue line, inside of membrane; pink line, outside of membrane. (C) Hydrophobicity of McLTPII.9, determined by ExPASy. y-axis, hydrophilicity of amino acids. (D) Predicted signal peptide in McLTPII.9. The predicted cleavage site was located between amino acid residues 26 and 27.


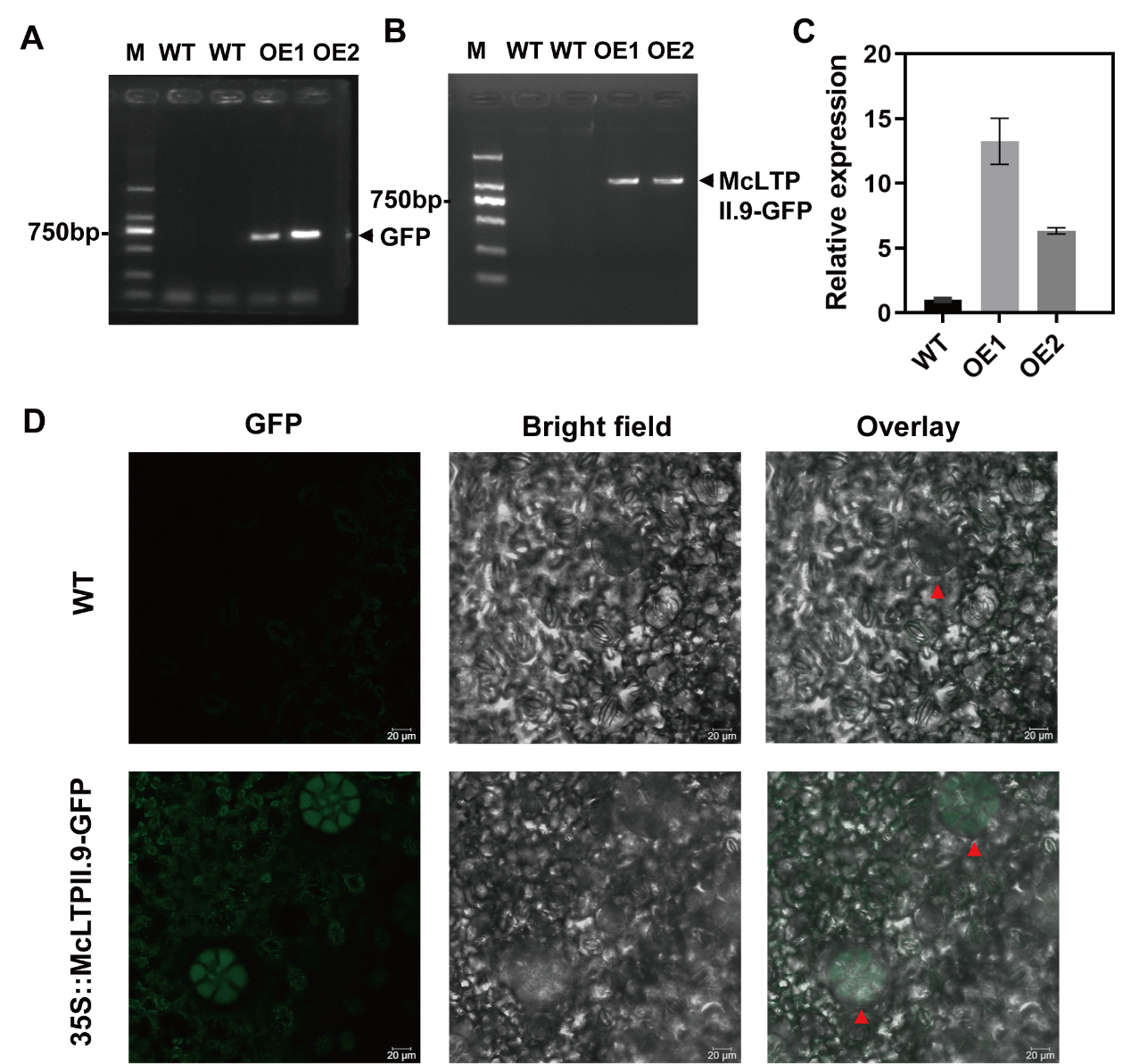


**Supplementary Figure S2. Identification of *McLTPII.9*-overexpressing transgenic peppermint.** (A) PCR products (720 bp) of genomic DNA extracted from wild-type (WT) and transgenic peppermint (OE1, OE2), which were amplified using GFP fragment primers. M, DNA marker (DL2000). (B) PCR products (1002 bp) of genomic DNA extracted from WT and transgenic peppermint, which were amplified using McLTPII.9-GFP fragment primers. M, DNA marker (DL2000). (C) RT-qPCR analysis of *McLTPII.9* expression in WT and transgenic peppermint. *MpActin1* was used as an internal control. (D) Localisation of McLTPII.9-GFP in mint leaves. Top row, no green fluorescence in WT peppermint. Bottom row, McLTPII.9-GFP transgenic peppermint. Bars, 20 μm. Red arrows indicate the peltate glandular trichomes.


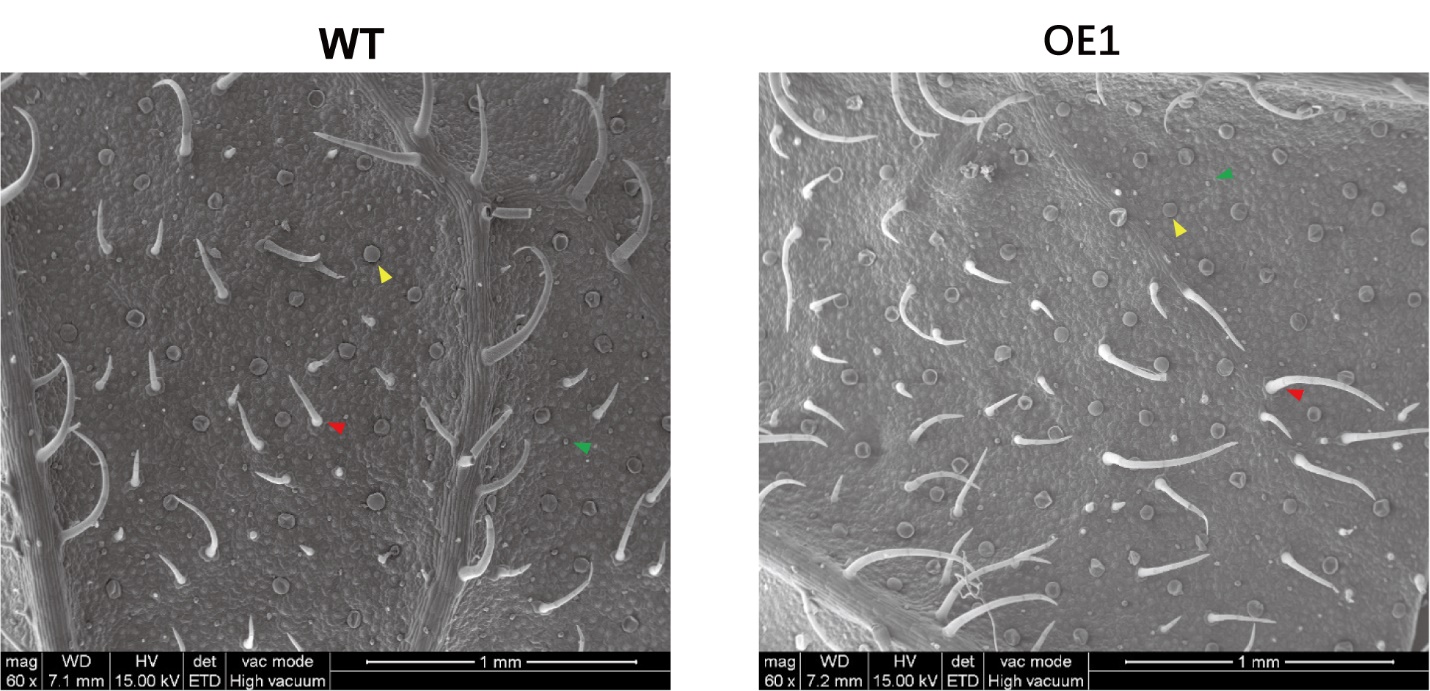


**Supplementary Figure S3**. **SEM observation of peltate glandular trichomes on the abaxial side of WT and *McLTPII.9-*overexpressing peppermint (OE1) leaves.**


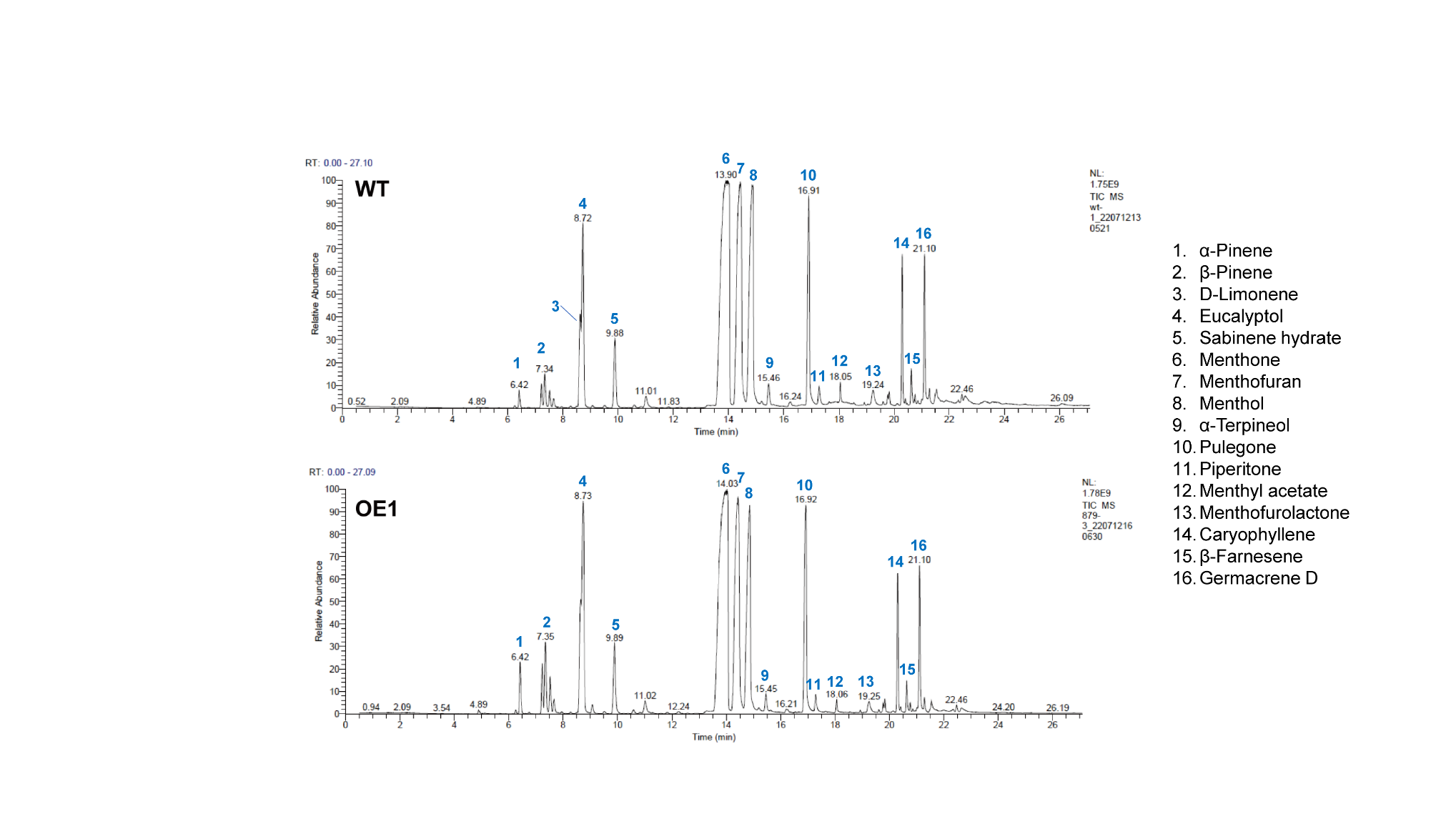
Yellow arrows indicate peltate glandular trichomes; red arrows indicate non-glandular trichomes; green arrows indicate capital trichomes. Bars, 1 mm.

**Supplementary Figure S4. Total ion chromatograms of volatile oil composition in wild-type and McLTPII.9-overexpressing peppermint.**

Some of the key terpenoid peaks were labeled with numbers that corresponding to the compounds.
